# Speciation of Arsenic in Medium Containing Bacterial Strains of *Lysinibacillus boronitolerans* and *Bacillus cereus*: Mechanism of Arsenic Removal

**DOI:** 10.3390/ijerph22111675

**Published:** 2025-11-04

**Authors:** Naidilene Chaves Aguilar, Adriele Santos Van Der Maas, Mayra Soares Santos, Rodrigo de Carvalho Hott, Márcia Cristina da Silva Faria, Bruno Lemos Batista, Cleide Aparecida Bomfeti, João Paulo de Mesquita, Jairo Lisboa Rodrigues

**Affiliations:** 1 Institute of Science, Engineering and Technology (ICET), Federal University of the Jequitinhonha and Mucuri Valleys (UFVJM), Teófilo Otoni 39803-371, Minas Gerais, Brazil; naidilene@hotmail.com (N.C.A.); adriele.maas@ufvjm.edu.br (A.S.V.D.M.); mayrasoaresantos@gmail.com (M.S.S.); rrodhott@yahoo.com.br (R.d.C.H.); marcia.faria@ufvjm.edu.br (M.C.d.S.F.); cleide.bomfeti@ufvjm.edu.br (C.A.B.); 2 Centro de Ciências Naturais e Humanas, Universidade Federal do ABC, Santo André 09210-170, São Paulo, Brazil; bruno.lemos@ufabc.edu.br; 3 Faculdade de Ciências Exatas (FACET), Universidade Federal dos Vales do Jequitinhonha e Mucuri, Diamantina 39100-000, Minas Gerais, Brazil; joao.mesquita@ufvjm.edu.br

**Keywords:** bioremediation, mining, arsenic, bacteria, soil, speciation

## Abstract

Environmental issues have become increasingly critical and frequent in recent decades due to excessive population growth and intensified industrial and mining activities. Among the most concerning contaminants is arsenic (As), a toxic element associated with severe environmental and human health risks. This study aimed to investigate the bioremediation potential of the bacterial strains *Lysinibacillus boronitolerans* and *Bacillus cereus*, elucidating the mechanisms involved in arsenic transformation and removal under controlled conditions. The strains were cultivated in liquid medium containing known concentrations of As(III) and As(V), and the chemical forms of arsenic were analyzed using High-Performance Liquid Chromatography coupled with Inductively Coupled Plasma Mass Spectrometry (LC-ICP-MS). The production of exopolysaccharides (EPSs) and arsenite oxidase activity were also evaluated. Morphological and elemental analyses were performed using scanning electron microscopy with energy-dispersive spectroscopy (SEM-EDS). The bacterial strains exhibited significant 69.38–85.72% reductions in arsenic concentration and approximately 14–15% volatilization rates. No EPS production or arsenite oxidase activity was detected, suggesting alternative detoxification pathways. SEM-EDS analyses revealed intracellular accumulation of arsenic, while LC-ICP-MS speciation confirmed interconversion between As(III) and As(V), indicating the action of methylation-dependent detoxification and membrane transport mechanisms. The findings demonstrate that *L. boronitolerans* and *B. cereus* possess efficient arsenic resistance and transformation mechanisms, even without conventional enzymatic pathways. These strains show strong potential for use in sustainable bioremediation of arsenic-contaminated environments, particularly in regions affected by mining activities.

## 1. Introduction

Arsenic (As) is considered by the U.S. Department of Health’s Agency for Toxic Substances and Disease Registry (ATSDR) to be one of the most hazardous substances to health due to its high toxicity and ability to cause severe and irreversible damage [1,2,3].

Global arsenic contamination has been increasing significantly, associated with both natural sources, such as hydrothermal deposits, weathering of sulfide minerals such as arsenopyrite, and anthropogenic sources, especially mining activity [4,5,6,7,8]. This problem is particularly evident in regions such as Paracatu and Minas Gerais, where Brazil’s largest open-pit gold mine, the Morro do Ouro Mine, is located. Although it has low gold contents (0.4 to 0.6 g Au t^−1^), this mine has extremely high concentrations of arsenic, above 4000 ppm, representing environmental and public health risks [7,9,10,11].

The severity of this contamination is evidenced by the identification of worrisome levels of arsenic in the Córrego Rico, with significant variations between the seasons of the year (4.05 μg L^−1^ in summer and 72.4 μg L^−1^ in winter) [12]. Such findings indicate a significant trophic transfer and accumulation, which may increase the risks of exposure of local populations. Similarly, studies in different contexts, such as in artisanal gem mining in the Jequitinhonha Valley (MG), have also identified high exposure to heavy metals, including arsenic [13,14]. These studies reported genotoxic effects, oxidative stress, elevated plasma peroxide levels, and reduced catalase activity in chronically exposed workers, showing clear and persistent toxicological effects. Prolonged chronic exposure to arsenic is associated with cancers (skin, lung, kidney, liver, and bladder), cardiovascular, neurological, hematological, and respiratory diseases, as well as diabetes mellitus and impairments to children’s intellectual development [7,15,16,17,18,19].

In this scenario, studies that integrate environmental microbiology and public health are essential to understand and mitigate the risks of human exposure to arsenic, which is recognized as one of the major global contaminants of public health concern [20]. The application of resistant microorganisms capable of transforming and removing arsenic compounds from contaminated environments represents a sustainable and low-cost biotechnological strategy with a high potential for social impact [21]. This approach is particularly relevant in mining regions such as Paracatu, Minas Gerais, Brazil, where human exposure to toxic metals and their genotoxic and oxidative effects have already been widely documented [4,8,13]. Microbial bioremediation contributes not only to the recovery of degraded ecosystems but also to the direct reduction in the toxic load that reaches human populations through water and food, thus representing an important preventive measure for promoting collective health.

Although microbial arsenic transformation has been extensively studied in several genera such as *Pseudomonas*, *Acinetobacter*, and *Bacillus*, there is still limited understanding of the specific mechanisms operating in *Lysinibacillus* species. *L. boronitolerans*, in particular, remains poorly characterized regarding its genetic and biochemical pathways for arsenic detoxification. Similarly, the physiological versatility of *Bacillus cereus* in response to metalloid stress has been reported, but its role in arsenic redox transformation and volatilization remains unclear. Therefore, investigating these two strains fills an essential gap in the current knowledge about bacterial adaptation and arsenic biotransformation in mining-impacted environments.

In addition to direct contamination by mining, foods such as rice stand out as critical sources of arsenic exposure due to their ability to accumulate this element in high concentrations. To address these challenges, recent research has explored sustainable technological strategies, such as using silver arsenate (Ag_3_AsO_4_) as an efficient photocatalyst in removing emerging contaminants, including endocrine disruptors, from contaminated waters [22]. At the same time, advanced chemical speciation techniques, such as LC-ICP-MS, have contributed significantly to the identification of resistant microorganisms capable of biotransforming toxic elements such as arsenic into less poisonous chemical species, demonstrating great potential for practical applications in environmental bioremediation [23,24,25,26,27,28,29].

In view of this scenario, this work aims to identify the biotransformations performed by microorganisms in chemical species of arsenic, using the speciation of samples by LC-ICP-MS coupling, aiming to obtain less toxic forms to the environment. It also seeks to evaluate the potential of these organisms for application in sustainable bioremediation techniques, contributing to significantly reducing the environmental and human health impacts in areas contaminated by arsenic [3,8,30,31].

## 2. Materials and Methods

### 2.1. Study Area, Sampling, and Bacterial Isolation

Soil samples were collected from five georeferenced points along the Rico Stream in Paracatu, Minas Gerais, Brazil, covering the gold-mining area of Morro do Ouro and downstream rural regions (17°13′5″ S, 46°53′56″ W to 17°14′59″ S, 46°51′38″ W). Sampling was carried out in March and August 2013 at a depth of 0–20 cm, and the material was transported under refrigeration to the Environmental Microbiology Laboratory (UFVJM). Total arsenic concentrations ranged from 344 to 1668 µg g^−1^, determined by acid extraction USEPA 3051 (U.S. Environmental Protection Agency, Washington, DC, USA) and quantified using ICP–MS (PerkinElmer NexIon 300D, Waltham, MA, USA). A 0.5 g soil aliquot from each site was inoculated into Luria–Bertani (LB) medium supplemented with 500 mg L^−1^ sodium arsenite (NaAsO_2_) and incubated at 30 °C for 14 days. Enriched broths were streaked on LB agar containing the same arsenic concentration and incubated for 72 h at 30 °C. Distinct colonies were purified, morphologically examined under a Zeiss MZ-8 (Carl Zeiss Microscopy GmbH, Jena, Germany), stereomicroscope, and preserved at −80 °C in 30% glycerol (*v*/*v*) for further analysis.

### 2.2. Determination of Bacterial Resistance and Molecular Identification

Isolates were cultivated in liquid LB medium (pH 6.8) under 100 rpm orbital shaking at 30 °C for 24 h, reaching approximately 1 × 10^8^ cells mL^−1^. The Minimum Inhibitory Concentration (MIC) was determined on solid LB medium containing 500–3000 mg L^−1^ NaAsO_2_, incubated for 72 h at 30 °C. MIC was defined as the lowest concentration that inhibited visible bacterial growth. All assays were performed in triplicate, including controls without arsenic. The most resistant strains (P1C1Ib, P2Ic, and P2IIIb) were further tested in liquid LB containing 0–3000 mg L^−1^ arsenic for 72 h at 28 °C and 100 rpm, with growth monitored at 600 nm. Genomic DNA was extracted, and the 16S rRNA gene was amplified using primers 5′-AAACTCAAATGAATTGACGG-3′ and 5′-ACGGGCGGTGTGTAC-3′. PCR conditions included an initial denaturation at 94 °C for 5 min, followed by 35 cycles (94 °C for 1 min, 55 °C for 1 min, 72 °C for 2 min), and a final extension at 72 °C for 5 min. Sequencing was performed by PBI (Brazil), and sequences were compared to the GenBank (NCBI) database for taxonomic identification.

### 2.3. Arsenic Accumulation and Metabolic Transformation Assays

The three selected strains were cultured in LB medium containing 750 mg L^−1^ sodium arsenite (III) or sodium arsenate (V) at 28 °C, 100 rpm, for 72 h. After incubation, cultures were centrifuged (10,000 rpm, 20 min, 4 °C), and pellets were washed three times with deionized water. Arsenic content in supernatants and biomass was quantified by ICP–MS (PerkinElmer Inc., Waltham, MA, USA) with a detection limit of 0.01 µg L^−1^, and results were expressed as percentage of removal and bioaccumulation. Enzymatic activity related to arsenic transformation was evaluated by adding 20 µL of 0.01 mol L^−1^ KMnO_4_ to 1 mL of bacterial culture. A pink coloration indicated oxidation of As(III) to As(V), while a yellow coloration indicated reduction of As(V) to As(III). All assays were conducted in triplicate, with sterile medium as a negative control.

### 2.4. Microscopic Analyses (AFM and SEM–EDS)

Morphological and elemental analyses of bacterial cells exposed to arsenic were performed using Atomic Force Microscopy (AFM; Park Systems Corp., Suwon, South Korea) and Scanning Electron Microscopy coupled with Energy-Dispersive X-ray Spectroscopy (SEM–EDS; Carl Zeiss Microscopy GmbH, Jena, Germany). Cells were collected by centrifugation (5000 rpm, 10 min), washed twice with ultrapure water, and air-dried. Samples were mounted on aluminum stubs with carbon tape and coated with a thin gold layer to ensure conductivity.

SEM–EDS analyses were conducted using a [model] microscope operating at 15 kV. Secondary and backscattered electron modes were used to observe cell morphology and intracellular arsenic accumulation. Elemental spectra were acquired by EDS (detection limit ~0.1 wt%) in both point and mapping modes to confirm arsenic presence. AFM imaging was conducted using a [model] instrument in contact mode at 25 °C, with a scan area of 5 μm × 5 μm. Surface topography and roughness parameters were evaluated before and after arsenic exposure to assess morphological alterations induced by metal stress.

### 2.5. Exopolysaccharide (EPS) Production

EPS production was assessed for *Lysinibacillus boronitolerans* (P1C1Ib, P2Ic) and *Bacillus cereus* (P2IIIb) cultured in liquid LB medium supplemented with 3.0 g L^−1^ NaAsO_2_ or Na_3_AsO_4_. Cultures were incubated for 72 h at 30 °C and 100 rpm, then centrifuged (13,000 rpm, 30 min, 4 °C). Equal volumes of ice-cold ethanol (1:1 *v*/*v*) were added to the supernatants, and the mixture was stored at 4 °C for 24 h to precipitate the EPS. The precipitate was washed, air-dried, and weighed to determine EPS yield (mg mL^−1^ of culture). All tests were performed in triplicate, including controls without arsenic, to evaluate the induction of EPS synthesis in response to metal stress.

### 2.6. Chemical Speciation of as in the Presence of Bacteria Using LC-ICP-MS Coupling

Arsenic speciation was performed using High-Performance Liquid Chromatography (HPLC, Flexar, PerkinElmer, Shelton, CT, USA) coupled to Inductively Coupled Plasma Mass Spectrometry (ICP–MS, NexION 300D, PerkinElmer, Shelton, CT, USA). Before each analysis, the HPLC system was rinsed with ultrapure water, and all filters were cleaned to prevent cross-contamination. Samples (100 μL) were injected under isocratic conditions at 25 °C. The mobile phase consisted of 10 mM HPO_4_^2−^/H_2_PO_4_^−^ buffer (pH 8.5) containing 2% (*v*/*v*) methanol, with a flow rate of 1.0 mL min^−1^, using a Hamilton PRP-X100 anion-exchange column (5 μm, 150 mm × 4.6 mm).

Quantification was performed by external calibration based on peak area integration. Calibration standards (2–20 μg L^−1^) were prepared daily by serial dilution of 2% (*v*/*v*) MeOH and 0.4% (*v*/*v*) HNO_3_, yielding calibration curves with R^2^ > 0.999. Rh^103^ (10 μg L^−1^) and Ga^69^ (0.5 μg L^−1^) were used as internal standards to ensure instrument stability. Each analytical sequence included method blanks and arsenic-free controls. All results are presented as mean ± standard deviation (SD) from three independent replicates. Statistical differences among treatments were assessed using one-way ANOVA followed by Tukey’s post hoc test at a significance level of *p* < 0.05.

The detection limits for these species were as follows: As(III) with LOD 0.02 µg L^−1^ and LOQ 0.07 µg L^−1^; As(V) with LOD 0.10 µg L^−1^ and LOQ 0.33 µg L^−1^; MMA with LOD 0.04 µg L^−1^ and LOQ 0.13 µg L^−1^; and DMA with LOD 0.06 µg L^−1^ and LOQ 0.20 µg L^−1^.

Table 1 summarizes the operating conditions of the technique employed for chemical arsenic speciation in bacterial strain samples using the LC_ICP_MS technique.

## 3. Results and Discussion

### 3.1. Evaluation of Exopolysaccharide (EPS) Production by Isolated Bactéria

The study strains were grown and then centrifuged to separate cells from the supernatant, which was produced after centrifugation. This, in turn, was treated with an iced alcohol mixture, which should promote EPS precipitation if present. Strains were irrelevant for EPS production, and no precipitate was observed at the end of the experiment.

The amount of EPSs produced by microorganisms is closely related to the specific conditions of cell growth and the composition of the culture medium [30,32]. Regarding the production of exopolysaccharides for both species under study, the isolation of exopolysaccharides for *Bacillus cereus* GU 812,900 was observed in stainless steel test panels that revealed a direct relationship between the corrosion rate and the EPS concentration produced by the species. Studies with Pb, Cu, and Zn adsorption on polysaccharides are also found in the literature; however, they were made by bacilli of another species, such as *Bacillus firmus*. The adsorption of metal ions is significantly affected by pH, initial concentrations of metal ions, polysaccharides, and the presence of other ions in solution. Pb, Cu, and Zn consumption reach 98.3% at optimum pH. The removal of metal ions is lower at neutral pH, and the initial adsorption rate is generally faster. The capture process obeys the isotherms of Langmuir and Freundlich. However, no results related to EPS production regarding arsenic resistance were found [33].

There are no reports of studies in the literature on EPS production in response to metal resistance by bacteria of the species *Lysinibacillus boronitolerans*, which is in line with the results of our work, since the isolates P1C1Ib, P2IIIb, and P2Ic are not efficient in terms of the production of exopolysaccharides.

### 3.2. Arsenic Oxidation by Bacterial Isolates

The test to identify the potential oxidation or reduction by specific enzymes, arsenite oxidase and arsenate reductase, should produce pink and yellow medium staining for positive results after KMnO_4_ treatment for their presence. After experiments, it was impossible to visualize an alteration in the culture medium color, making it impossible to identify the presence of the enzymes. Furthermore, no oxidation of arsenite to arsenate was observed in the abiotic control (medium without bacterial inoculation), confirming that the redox changes observed in the experimental treatments are exclusively of biological origin. However, it was verified in the literature that the description of the action of the enzyme arsenate reductase in the *Bacillus cereus* AG-27 strain, which is related to the presence of a gene called arsC [34]. For *L. boronitolerans*, no studies were found in the literature to verify the presence of these enzymes.

Arsenic resistance mechanisms involving the production of EPSs and the action of the enzyme arsenite oxidase were not found for the three strains classified as *B. cereus L. boronitolerans* analyzed in this work. However, these strains can grow in medium containing high concentrations of arsenite (23.09 mmol L^−1^), making it clear that there are mechanisms of cellular action for this resistance that have not yet been evaluated.

Moreover, even if they belong to the same species, the strains behave differently and, therefore, other arsenic resistance mechanisms will be evaluated in the future, since these three strains have great potential to be applied in environmental bioremediation processes.

### 3.3. Images Obtained by AFM and Percentage of As in Bacteria by SEM–EDS

**Figure 1:**
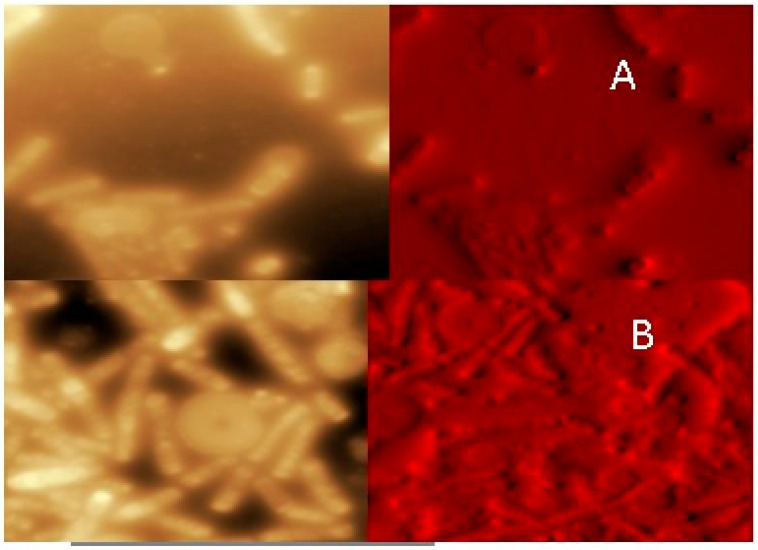
Contact mode AFM images of a mica surface with P2IIIB strain (*Lysinibacillus boronitolerans*) cultivated in the absence of As III (**A**) and presence of As III (**B**).

Figure 1 shows the bacterial strains identified by AFM in medium with the absence and presence of As [33]. Each bacillus has an average size of 150 μm. There was no change in the size of the bacillus in the presence and absence of arsenic.

**Figure 2:**
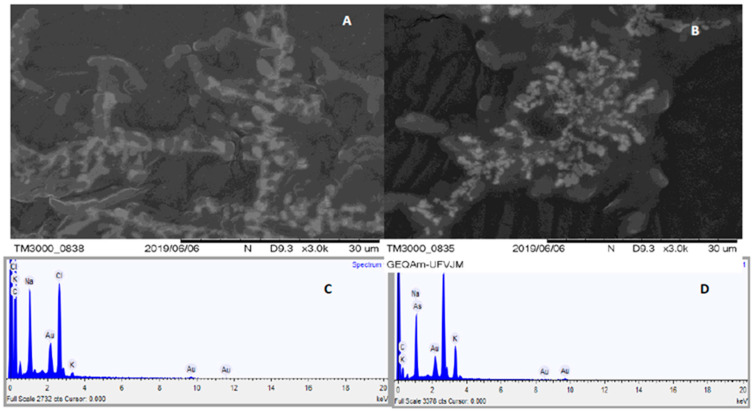
SEM-EDS images of bacteria grown in the absence (**A**,**C**) of As and in the presence of As (**B**,**D**).

Figure 2 presents a graph showing the percentage of arsenic detected inside bacterial cells cultivated with and without arsenic exposure.

It is clearly observed that the cultivated bacterium in an environment that contained As could absorb the As from this environment. At the same time, they showed a high concentration of As in their interior (2-D) [35,36]. Such confirmation is made by the percentage of arsenic identified by the EDS methods. This fact provides evidence and demonstrates the mechanism by which these bacteria absorb As. As a negative control, you can observe that the cultivated bacteria in an As-free environment (2-A) did not show As percentage in their interior (2-C), through the MEV-EDS techniques, which confirms the proposed mechanism.

Previous studies showed a clear difference in the oxidation state of the absorbed by the strains. Chemical abiotic processes predominantly produce the absorption of As(V), while the bacterial precipitation process leads to the absorption of As(III) and As(V). As(III) is proposed to result from the subsequent reduction of arsenate to arsenite by bacterial activities [18,37,38,39,40,41].

### 3.4. Chemical Speciation of as in the Medium Containing the Studied Bacteria Using LC-ICP-MS Coupling

The reduction in As concentration in the different forms of toxicity evaluated for P2Ic, P2IIb, and P1C1Ib strains was estimated between 69.38% and 71.88% for arsenite and between 82.39% and 85.72% for arsenate. The reduction in As concentration in media where the identified strains were placed for growth shows their essential role in bioremediation processes. However, it is necessary, besides quantifying the reduction of the toxic metal, to specify the As remaining in the post-culture medium of the bacteria, to understand which form of biotransformation is used by the strains as a defense mechanism. By identifying the mechanism, it can be manipulated to use environmental remediation and remediation techniques better.

**Figure 3:**
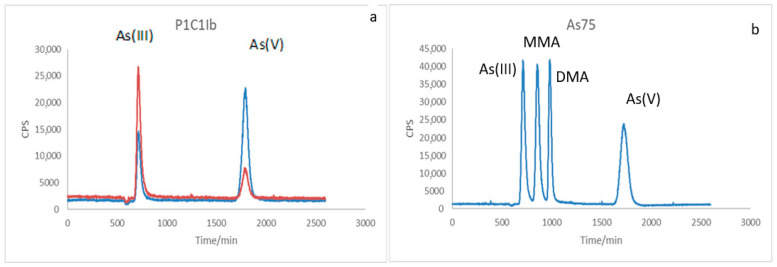
LC–ICP–MS chromatograms showing arsenic speciation for the *Lysinibacillus boronitolerans* strain (P1C1Ib). (**a**) Chromatographic profile of post-cultivation samples grown in arsenate (blue line) and arsenite (orange line) media. The retention times correspond to the peaks of As(III) (~2.5 min) and As(V) (~4.2 min), indicating interconversion between the two species during bacterial growth. (**b**) Standard chromatogram for As species (As(III), MMA, DMA, As(V)) at 20 µg L^−1^. Detector response is expressed in counts per second (CPS) as a function of retention time (min).

**Figure 4:**
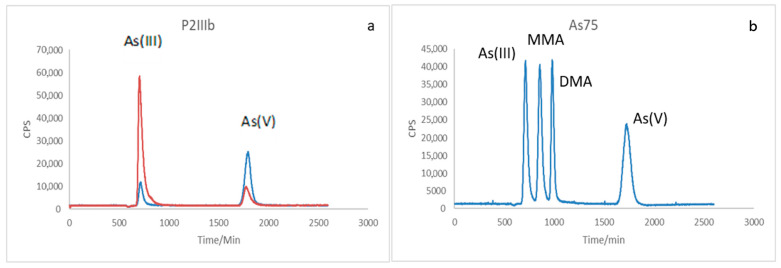
LC–ICP–MS chromatograms showing arsenic speciation for the *Bacillus cereus* strain (P2IIIb). (**a**) Chromatographic profiles of post-cultivation samples grown in arsenate (blue) and arsenite (orange) media. Peaks at approximately 2.5 and 4.2 min correspond to As(III) and As(V), respectively, indicating bidirectional redox transformation during incubation. (**b**) Standard chromatogram of As species (As(III), MMA, DMA, As(V)) at 20 µg L^−1^. Signal intensity expressed in counts per second (CPS) versus retention time (min).

Figure 3a and Figure 4a show the speciation of As present in the post-cultivation sample of strain P1C1Ib and P2IIIB, in blue, arsenate cultivation, and in orange, arsenite cultivation, the pattern of the chemical forms of arsenic, represented in Figure 3b and Figure 4b. Comparing the samples with the standard, it is clear that both As(III) and As(V) appear in the two samples analyzed. In the Blue sample, the appearance of As(III) was observed even when the medium only contained arsenate, and the opposite, in the orange sample, the appearance of As(V) was observed when enriched with only arsenite in the medium.

One hypothesis is that the bacteria used biotransform arsenic as a defense mechanism in detoxification, because although arsenite is more toxic than arsenate, it can be methylated to monomethylarsonic acid (MMA) or dimethylarsinic acid (DMA) and subsequently volatilize the metalloid through this enzymatic methylation [7,40].

In this detoxification pathway, organisms absorb arsenate through the phosphate transport system, while arsenite is captured through aquaglyceroporins, multifunctional channels that carry solutes such as glycerol and urea. Within the cell, arsenate is reduced to arsenite by the enzyme ArsC. It is then expelled from the cytoplasm through an arsenite carrier protein, ArsB, which uses membrane potential energy, sequestered in intracellular compartments such as free arsenite, along with glutathione (GSH) or other thiols [34].

Some proteins act to expel arsenite to the external environment after biotransformation, such as ArsB and ArsD, which act as regulatory repressors [36]. ArsR is a repressor that controls basal expression levels in response to the presence of arsenite. In contrast, ArsD is an arsenite transferring protein metallo to ArsA, which in turn is an ATPase that forms a complex with the ArsB transmembrane protein to expel the arsenite from the cell [17,35,40,41].

Comparing the two strains, it can be seen from the concentration of arsenic remaining in the samples that strain P1C1Ib has a higher potential for decontamination than P2IIIb, since, starting from the two bacteria of the same initial arsenic concentration in the culture medium, P1C1Ib presented lower arsenic concentration at the end of the experiment, and may have biomethylated and volatilized part of it, which removes the species from the sample, as well as bioaccumulated by precipitation in the cell or by complexation in the cell surface.

### 3.5. Detoxification Mechanism Through Arsenic Volatilization

The arsenic detoxification mechanism was studied by observing the final concentration of the metal present in the culture medium, where the bacteria were placed to grow. Initially, the strains were incubated in arsenate medium, and the arsenite procedure was repeated separately, simulating the contaminated environment to verify their tolerance and growth potential. After growth of the strains, they were removed from the medium, and the same was taken for quantification and speciation of the remaining arsenic.

At the end of the test, chemical speciation of arsenic forms was made in the environment where the bacteria were incubated. The chemical speciation of the remaining species in the medium was also made after incubation. The percentage of each species and the percentage lost by the volatilization mechanism were calculated from the remaining concentration.

Each bacterial strain was evaluated under two experimental conditions: one in a medium initially containing arsenite [As(III)] and another with arsenate [As(V)] as the predominant arsenic species. Accordingly, the two rows presented for each strain in Table 2 correspond to these distinct initial oxidation states, allowing the comparison of transformation efficiency and volatilization behavior under both conditions. It can be observed in Table 2, that around 14% of As is volatilized, with a greater proportion being converted As(V) to As(III).

These results agree with the literature. The detoxification mechanism converts As (V) to As (III) to form methylated species later and then promotes As volatilization, thus reducing its concentration in the medium [35]. Thus, volatilization loss can be confirmed as one of the mechanisms of As elimination by the bacteria P1C1Ib and P2IIIb. Similar work was performed with cyanobacteria, as presented [11] on arsenic biotransformation in a mining area.

These results reinforce not only the efficiency of the studied strains but also their potential applications in environmental and public health contexts. The observed transformation and volatilization capacities of *Lysinibacillus boronitolerans* and *Bacillus cereus* indicate a biological detoxification mechanism that can be explored in sustainable strategies for bioremediation of contaminated soils and waters. In mining regions such as Paracatu, Minas Gerais, Brazil, where elevated arsenic concentrations in water bodies and genotoxic effects in exposed populations have been reported [4,8,13], the application of these microorganisms may represent a low-cost and effective alternative to reduce arsenic mobilization and prevent its bioaccumulation in the food chain. Therefore, the findings of this study contribute not only to advancing knowledge of microbial resistance and metal transformation mechanisms but also to developing environmental technologies aligned with preventive public health principles and the United Nations Sustainable Development Goals (SDGs 3 and 6) of the 2030 Agenda.

## 4. Conclusions

This study demonstrated that *Lysinibacillus boronitolerans* and *Bacillus cereus* can transform, accumulate, and volatilize arsenic species under controlled laboratory conditions. Analyses performed by Liquid Chromatography coupled with Inductively Coupled Plasma Mass Spectrometry (LC–ICP–MS), Scanning Electron Microscopy coupled with Energy-Dispersive X-ray Spectroscopy (SEM–EDS), and Atomic Force Microscopy (AFM) confirmed the redox interconversion between As(III) and As(V), intracellular accumulation of arsenic, and volatilization of approximately 14–15% of the total content. No production of extracellular polymeric substances (EPSs) or oxidase/reductase activity was observed, indicating that detoxification occurs predominantly through intracellular reduction and efflux mechanisms rather than extracellular complexation.

The results of this study expand the current understanding of bacterial mechanisms involved in arsenic resistance, particularly within the genus *Lysinibacillus*, which remains scarcely explored in this context. These findings enhance the knowledge of microbial processes driving arsenic biotransformation and support the potential application of these strains in the bioremediation of contaminated environments.

Future studies integrating genomic, proteomic, and transcriptomic approaches, such as whole-genome sequencing, LC–MS/MS proteomics, and RNA-Seq, are encouraged to elucidate further the molecular and regulatory pathways underlying arsenic detoxification and volatilization in these bacteria.

## Figures and Tables

**Table 1:** Liquid chromatography and ICP-MS operating conditions for tissue samples’ total arsenic determination and speciation analysis.

**LC Conditions**	
Column	Hamilton PRP-X100 (5 μm, 150 mm, 4.6 mm)
Mobile phase	10 mM HPO^−2^_4_/H_2_PO _4_, pH 8.5, 2% (*v*/*v*) MeOH
Mobile phase flow rate	1 mL min^−1^
Oven temperature (column)	25 °C
Equilibrium	1 min
Run	9.0 min
Wash	1 min
Mode	Isocratic
Injection volume	100 μL
Measurement	Peak area
**ICP-MS experimental conditions**	
Radiofrequency power	1200 W
Scan mode	Peak hopping
Gas flow rates	Plasma 15 L min^−1^; auxiliary 1.2 L min^−1^
Nebulizer gas flow	0.86–0.98 L min^−1^
Internal standards (1)	Rh^103^ (10.0 mg/L) for total As determination
Internal standards (2)	Ga^69^ (0.5 mgL^−1^ for As speciation [ICP-MS’s peristaltic pump is set at 10 rpm (0.5 mL min^−1^)] for As speciation
Interface	Platinum cones
Sampler	1.1 mm
Skimmer	0.9 mm
Resolution	0.7 amu
Isotope^75^As	

Source: Author data.

**Table 2:** Distribution of arsenic species after bacterial incubation. For each strain (P1C1Ib and P2IIIb), the first line refers to the culture initially containing As(III) and the second to As(V). Values represent the percentage of each species remaining in the medium and the estimated volatilized fraction.

Bacterial Strain	As III	As V	As Volatilized
P1C1Ib	27%	59%	14%
P1C1Ib	76%	9%	15%
P2IIIb	70%	16%	14%
P2IIIb	24%	61%	15%

Source: Author data.

## Data Availability

The data presented in this study are available on request from the corresponding author.
